# Pollen viability and longevity in *Juniperus* taxa native to Slovakia

**DOI:** 10.1038/s41598-024-53152-7

**Published:** 2024-02-14

**Authors:** Martin Galgóci, Andrej Kormuťák, Miroslav Klobučník, Dušan Gömöry, Ivan Lukáčik, Peter Boleček

**Affiliations:** 1 Plant Science and Biodiversity Center, Institute of Plant Genetics and Biodiversity, Slovak Academy of Sciences, Akademická 2, P.O. Box 39A, 950 07 Nitra, Slovak Republic; 2 Faculty of Forestry, Technical University in Zvolen, T.G. Masaryka 24, 960 53 Zvolen, Slovak Republic; 3 Faculty of Natural Sciences, Constantine Philosopher University in Nitra, A. Hlinku 1, 949 74 Nitra, Slovak Republic

**Keywords:** Evolution, Genetics, Plant sciences

## Abstract

Pollen viability, dispersion ability and longevity during deep-freezer storage were studied in three *Juniperus* taxa distributed in Slovakia. All these characteristics of pollen are closely related to the pollination and/or fertilization success of the junipers in nature. Pollen viability varied considerably between the three populations of *J. communis* and one population of each, *J. sibirica* and *J. communis* var. *intermedia*. Pollen germination rate ranged between 40.75% and 75.06%, and pollen tube length between 30.32 and 40.41 µm. A clear tendency indicates a higher germination rate of *J. communis* pollen from lower altitudes and reduced germination of *J. sibirica* and *J. communis* var. *intermedia* pollen from higher altitudes. The dispersion potential of the *J. communis* pollen during its shedding seems relatively low. In 2021, pollen cloud density was diluted at 68.1% at the 4 m distance from the test shrub, in 2022 of 52.1% at the 17 m distance from the pollen source. A deep-freezer storage of juniper pollen in a double-walled polyethylene bag with silica gel was not efficient enough, as indicated by the drop of pollen germination rate of 31.2% in *J. communis* and of 79.4% in *J. sibirica* during a 1-year storage period at − 81 °C.

## Introduction

Among 76 Juniperus species recognized so far, the species *J. communis* hast he largest distribution area spreading from Europe to Armenia and to central Russia^1^. In Slovakia, the species is growing on the entire territory of the country. It may be found from the lowlands up tot he mountain zone, along with the two additional taxa, i. e. *J. sibirica* and *J. communis* var. *intermedia* which occur naturally at the higher altitudes of the Carpaticum region^2,3^. The last mentioned taxon is supposed to be a hybrid between *J. communis* var. *communis* and *J. sibirica*^3^. All the three Juniperus species fulfill important ecological function in the nature serving as the pioneer trees which initiate the early stages of secondary succession of the forests on devastated pasture lands of the hills and mountains^3,4^. Junipers are dioecious species with the sexes occurring on different shrubs. Although they reproduce prevailingly by sexual means, little is known about the biology of their sexual reproductive cycle^5^. At the pollen level, most information comes from the studies on pollen morphology and mechanism of in vitro pollen germination. The main outcome of a comparative study on pollen shape and sculpture in 18 species of the family Cupressaceae, including 5 juniper species, is generalization that uniform morphology and surface structure of the pollen is an indication of the homogeneity of the entire family^6^. Particular architecture of the pollen surface determines the taxoid type of pollen hydration during pollen germination that is characteristic of Cupressaceae, Taxaceae and Taxodiaceae. The exine pore was shown to play a decisive role in this process, enabling penetration of water into the intine, its subsequent expansion and rupture of the exine from the pollen grain, enabling pollen tube growth. The details of the initial stages of pollen germination in *Cupressus arizonica* and *J. communis*, as well as the entire process of pollen germination of the latter under in vitro conditions and in situ, were also illustrated cytologically^7–10^. In spite of chronically acknowledged low quality of *J. communis* seeds on European continent^5,7,11,12^, an attempt has yet to be made so far to relate this phenomenon to the pollen viability. Alexander's stain was applied on the pollen samples of *J. thurifera*, *J. sabina* and their putative hybrid to follow pollen hydration during their study on morphology and size of the pollen grains. However, an attempt had yet to be made to test pollen viability^13^. The only report dealing with *J. communis* pollen viability refers to the effect of mineral fertilization on plant growth, production of male cones and pollen quality in *J. communis* species, providing the first data on germination potential of the pollen as revealed by in vitro germination test^14^. These data were obtained from sixty plants derived from the same parental line and grown under experimental conditions, which may hinder the exact evaluation of pollen viability at the species level. Therefore, the subsequent attempt we have made in the present study aims for a more precise evaluation of mature pollen viability in three *Juniperus* taxa ingenious to Slovakia using a representative sample of the individuals of each taxa from their natural habitats. The study was paralleled by testing of pollen longevity in *J. sibirica* and *J. communis* under deep-freezer storage to check the species-specific reaction to storage conditions, if any. It is considered that long-term storage of pollen makes possible hybridization between trees differing in flowering phenology and between the trees growing in remote places. Accordingly, the objective of the attempt was to retain the highest possible viability of the pollen for the period of 1 year at least, which is necessary for overcoming profound difference in flowerinf phenology of the species as a prerequisite for their reciprocal crossing. At the stage of shedding pollen, the dispersion capacity of *J. communis* pollen was estimated by tracing its presence in the field at the increasing distances from the test shrub. Both these experiments are believed to shed more light on the process of sexual reproduction of junipers under climatic conditions of Slovakia.

## Results

### Pollen viability of investigated juniper species and populations

**Figure 1:**
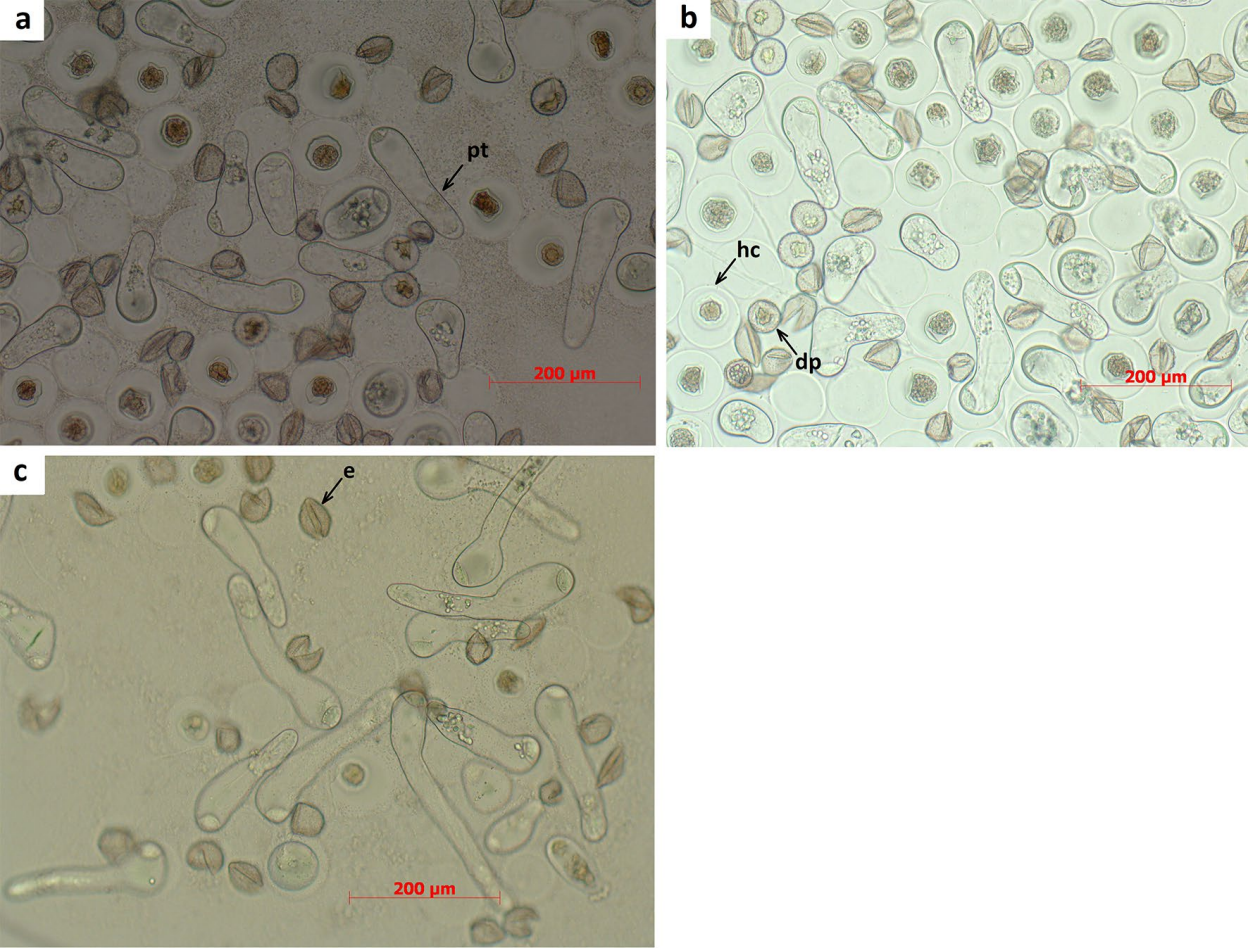
Germinating pollen of *J. communis* (**a**), *J. sibirica* (**b**) and *J. communis* var. *intermedia* (**c**); pt-pollen tube, hc-non-germinating pollen grain with hydrophilic capsule, dp-non-germinating pollen grain which failed to form hydrophilic capsule, e-exine.

Pollen viability in investigated juniper taxa and populations was shown to be relatively low, as evidenced by the pollen germination percentage ranging between 40.75% and 79.06% only and pollen tube length averaging within the 30.32 µm and 40.41 µm limits (Table 1). Within established variation pattern, the most conspicuous differences in pollen germination rate were revealed between the pair of *J. communis* populations Priechod and Cervená Skala, which occur at lower elevations and the putatively hybrid populations of *J. communis* in Besník and *J. communis* var. *intermedia* on Kralová Studna both of which are distributed at higher altitudes. The latter pair of populations was characterized by a lower germination rate of their pollen than populations from Priechod and Cervená Skala. The species *J. sibirica* from Kralová Hola Mtn. occupies an intermediate position in this respect, but according to Duncan's grouping, the species inclines by its pollen viability parameters to *J. communis* population from Cervená Skala rather than to *J. communis* var. *intermedia* on Kralová Studna. The outlined tendency may indicate that a low quantity of functional pollen at high elevations conditioned by the harsh weather on the mountains is a probable reason for the high percentage of empty seeds not only in Norway spruce^15^ but also in juniper species. The remarkable feature of *Juniperus* pollen germination is a pronounced variation between individual shrubs of a given taxon or population. For example, the difference between the minimal and maximal germination percentages in *J. sibirica* and *J. communis* individuals may be mentioned,ranging from 4 to 87% in the former and 6% to 92% in the latter. The same holds for the pollen tube length, which has varied to a lesser degree than the pollen germination percentage. The shortest tubes were recorded in *J. sibirica* and putative hybrid population *J. communis* from Besnik, the longest in the putatively hybrid taxon *J. communis* var. *intermedia* (Table 1). Nested ANOVA confirmed statistically significant differences in average pollen germination between investigated populations (F = 147.01***, DF = 4) and individual shrubs (F = 15.85***, DF = 59). Like germination rate, the differences in pollen tube length were highly significant between compared populations (F = 92.01***, DF = 4) and individual shrubs of a given population (F = 60.42***, DF = 45). Cytological examination of germinating pollen at the end of incubation period indicates that the fraction of non-germinating pollen involves not only the pollen grains with preserved exine on their pollen bodies but also those that have imbibed water, discarding subsequently their exines into cultivation medium but which failed finally to form the pollen tubes (Fig. 1). This modifies considerably the option which denies the ability of pollen grains to imbibe water from medium and subsequent formation of hydrophilic capsule by non-germinating juniper pollen^7^. Our data contradict this finding, indicating a large amount of pollen grains with hydrophilic capsules within a fraction of non-germinating pollen grains of juniper species. This part of the mechanism governing pollen germination requires additional verification.

**Table 1:** Pollen viability in three juniper species.

Species	Locality	Altitude (m)	Germination rate (%)]			Pollen tube length (µm)		
			N	Mean’ ± s.d	Duncans grouping	N	Mean ± s.d	Duncan’s grouping
*J. communis*	Priechod	530	45	79.06 ± 11.28	A	1.350	32.65 ± 953	C
*J. communis*	Cervená Skala	785	45	52.22 ± 19.26	B	1.350	34.75 ± 14.20	B
*J. sibirica*	Kralova Hola	1846	42	48.40 ± 20.63	BC	1.260	30.37 ± 13.40	D
*J. communis* var*. intermedia*	Kralova Studna	1284	15	44.46 ± 12.37	C	450	40.41 ± 18.36	A
*J. communis*	Besnik	994	45	40.75 ± 25.28	D	1.350	30.32 ± 14.57	D

### Dispersion of *J. communis* pollen

**Figure 2:**
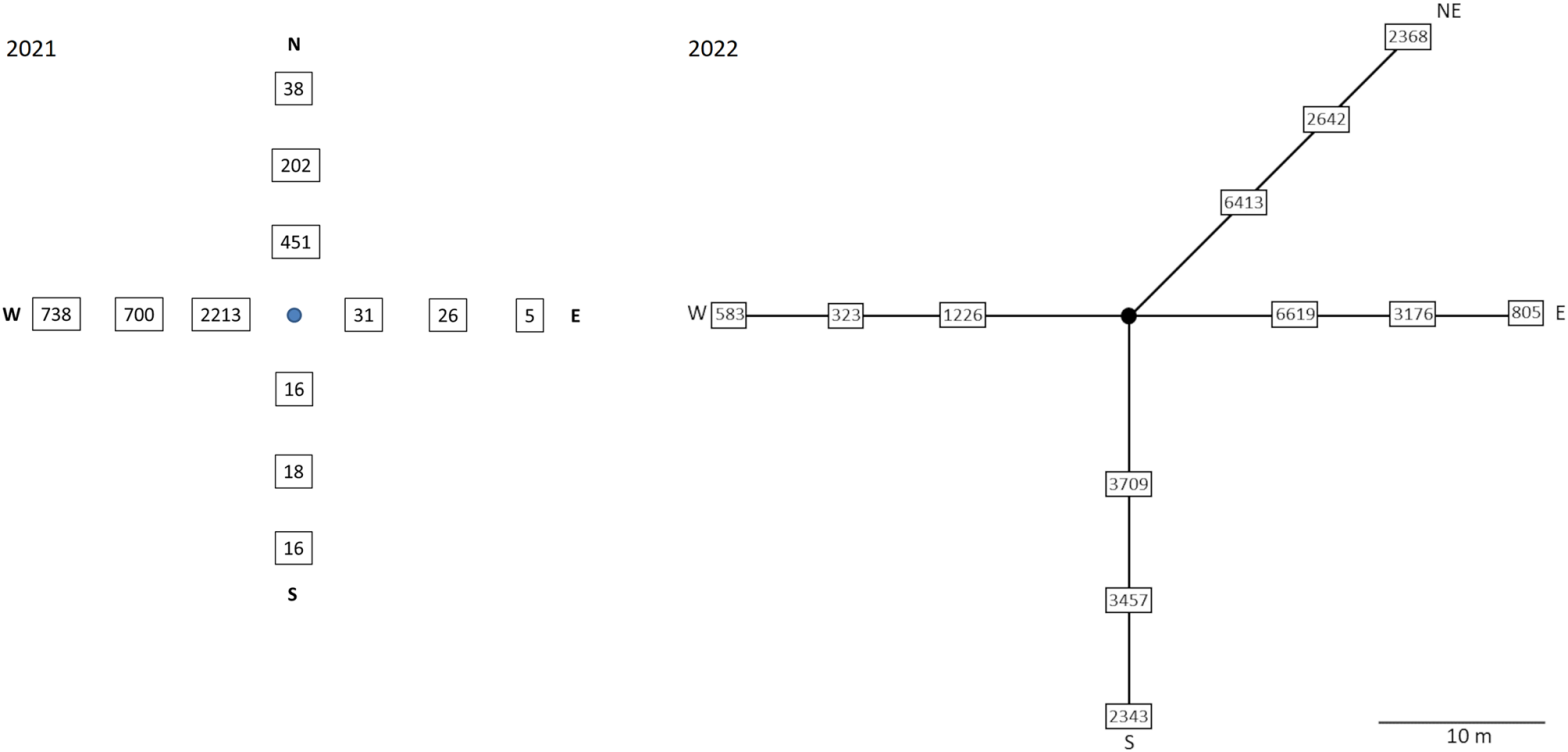
Number of pollen grains detected at the 2, 4 and 6 m distance from the male shrub in 2021 and at the 10, 17 and 24 m distance from the male shrub in 2022.

Pollen dispersion monitoring carried out during the two successive seasons on the example of one *J. communis* individual has provided the first insight into density and dispersion ability of the pollen grains around the male shrubs at the time of pollen shedding. The results diagrammed in Fig. 2 proved profound variation in the amount of pollen produced in the two respective years by the test shrub. The highest number of pollen grains scored in 2021 at the 2 m distance from the shrub was 2213, whereas, in 2022, it was 6619 pollen grains trapped on surface of the microscopic slide at the 10 m distance from the shrub. The above density of pollen cloud has been progressively diluted with increasing distance from the shrub. In 2021, the number of pollen grains in the cloud has diminished by 68.4%, as revealed in the prevailing direction of wind blowing. At a distance of 4 m from the shrub, this decline reached the level of 700 pollen grains. The comparable tendency was also confirmed in 2022 when the density of pollen cloud was diluted by 52.1% at the 17 m distance, reaching 3176 pollen grains, and an additional 74.7% at the 24 m distance from the shrub, reaching 805 pollen grains per score segment of the microscopic slide. Based on these data, a preliminary conclusion may be drawn postulating restricted dispersion ability of *J. communis* pollen grains.

### Viability of deep-freezer-stored pollen

A uniform response of *J. communis* and *J. sibirica* pollen towards storage conditions was only registered in the germination rate. In pollen tube length, both species behaved in different ways. In *J. communis*, the germination potential of stored pollen declined by 31.2%, whereas in *J. sibirica*, it was 79.4% compared to freshly collected pollen. The data presented in Table 2 shows that germination of 1-year stored pollen averaged in *J. communis* at 51.27% and in *J. sibirica* at 10.88%. In *J. sibirica*, the observed decline in germination percentage was also paralleled by the reduced length of pollen tubes, which lowered growth by 38.2% compared with fresh pollen. The exception was, in this respect, *J. communis* pollen, whose pollen tubes were after storage 9.4% longer than pollen tubes of fresh pollen.

**Table 2:** Pollen viability of freshly collected and one-year stored pollen of two juniper species.

Species	Pollen treatment	Germination rate (%)		Pollen tube length (µm)	
		N	Mean ± s.d	N	Mean ± s.d
*J. communis*	Fresh	18	74.50 ± 11.55	540	33.14 ± 9.94
	Stored	18	51.27 ± 18.02	540	36.55 ± 18.78
*J. sibirica*	Fresh	18	52.61 ± 16.00	540	29.49 ± 8.66
	Stored	18	10.88 ± 10.91	540	18.22 ± 13.25

## Discussion

All three pollen biology aspects treated in the present study directly connect with the widely acknowledged reproductive failure of junipers in nature. To illustrate this failure, the results of seed quality analysis in the UK should be mentioned based on 686 mature berries of common juniper, which contained only 1.96% of fully developed seeds^5,11^. Pollen viability of junipers is crucial in this context, providing necessary stimulus for the initial transformation of an ovule and its subsequent development into a mature seed. Domestic taxa of junipers were found to differ profoundly in this characteristic of their pollen. The populations of *J. communis* from lower altitudes have, as a rule, exhibited higher germination rates than *J. sibirica*, *J. communis* var*. intermedia* and *J. communis* populations from higher altitudes. The hybrid nature and supposed increase of meiotic irregularities in populations of *J. communis* on Besnik and *J. communis* var. *intermedia* on Kralová Studna is an additional factor which adversely affects the viability of their pollen. In absolute terms, the revealed germination potential of *Juniperus* taxa is not too high, spanning between 40.75% and 79.06%. The question of whether this amount of functional pollen is sufficient for effective pollination of the juniper needs further investigation. Only one study of this kind is available that makes a comparison of the juniper pollen germination possible^14^. The effect of mineral nutrition on male cone production and quality of mature pollen in *J. communis* has accordingly been studied using non-fertilized plants as a control. After 4-day cultivation of pollen in vitro, the germination percentage reached 24.9% level in the respective shrub, which is a much lower value than the data we obtained. This discrepancy is probably due to the different methodological approaches applied in the respective studies. In the study mentioned above, a liquid medium has, for example, been used for pollen cultivation instead of the solid agar medium we used. A shortened period of pollen incubation in the experiment with fertilization to 4 days is an additional explanation for this discrepancy. It was emphasized earlier that *J. communis* pollen requires a 6–7 day incubation period to express fully its germination potential under in vitro conditions^10^. Of no less importance than pollen viability is the successful reproduction of junipers in nature and the dispersion potential of their pollen, mainly because of the dioecious nature of juniper species. This pollen biology aspect has not been analysed in junipers yet. The preference has been given to seed dispersal in *J. virginiana* by birds^16^ and in *J. communis* by red squirrels^17^. A more complete survey on the subject has been provided recently^18^. As for the pollen dispersion, our data suggest a relatively rapid melting of pollen clouds after pollen release from microsporangia. In *J. communis,* the pollen cloud density was found to drop to 12% only at the 14 m distance from the pollen source. This supports the idea of a restricted dispersal ability of juniper pollen. The opinion prevails that it is low height of the shrubs which is responsible for the restricted dispersion of pollen around male individuals. Accordingly, the distribution of significant amount of pollen on long distances is improbable in juniper^10^. Within the context of pollen viability, the retaining of its germination potential during short- or long-term storage plays an important role. It enables breeders to overcome the differences in pollen shedding and ovule receptivity during controlled pollination of trees in the same locality or trees in widely separated regions^19^. On account of artificial crossing experiments, which are under progress between *J. communis* and *J. sibirica*, the storage of pollen was an inevitable step. Each species occupies the habitat at different altitudes, differing profoundly in the course of its phenology. Pollen storage is the only means which has enabled the testing of the genetic status and crossability relationships between the parental species in a reciprocal way. Unfortunately, our expectations relative to pollen quality after storage at − 81 °C have not been fulfilled. The decline of germination rate was profound in both species, reaching 31.2% in *J. communis* and 79.4% in *J. sibirica.* Likewise, the pollen tube growth in *J. sibirica* was reduced by 61.7% compared to fresh pollen. Compared with the results of *J. communis* pollen storage at − 20 °C for four months, when only negligible differences in pollen viability of fresh and stored pollen were registered^20^, the storage at − 81 °C over one year represents a rather adverse figure. Different storage duration periods are not the only reason for such an unexpected outcome. A double-walled polyethylene bag does not represent a simple solution for a deep-freezer storage of juniper pollen. It probably does not satisfy the requirements for maintaining constant humidity of the pollen during its long-term storage. With its 30% water content^21^, the mature pollen of Cupresaceae is difficult to store for a longer period of time without using a more sophisticated container instead of polyethylene bags. Based on practical experiences with long-term storage of Abies and Pinus pollen at − 20 °C using desiccator with silica gel^22–24^, it seems that juniper pollen is much more sensitive to storage conditions compared to pollen of firs and pines. Therefrore, the use of a standardized container with liquid nitrogen as a storage medium is offerred as the only alternative for *Juniperus* pollen.

## Conclusions

Pollen viability varies considerably in the three juniper species growing in the Middle Slovakia region. In particular, it is true of germination rate, which exhibits some tendency towards dependency on the habitat altitude. As a rule, the pollen of *J. communis* and its populations from a lower altitude has exhibited higher germination rates than the pollen of the species in the mountains. We are not on a firm ground on whether lowered germination potential of the putative hybrid population *J. communis* var. *intermedia* from Kralová Studna is due to its hybrid nature or if it is conditioned by the harsh climatic conditions. Additional studies on the course of meiosis are highly desirable to clarify the matter in more detail. Preliminary data on the dispersion of *J. communis* pollen support the idea of a restricted flow of the species' pollen under field conditions, which may be a possible explanation for the low quality of the *J. communis* seeds in Europe. An attempt to prolong the longevity of the juniper pollen by a deep-freezer storage using double-walled polyethylene bags has not met with success. A further improvement should be made in selecting a more appropriate pollen storage method based preferentially on a liquid nitrogen storage medium.

## Methods

### Plant materials

Three *Juniperus* species, naturally distributed in Middle Slovakia, were subjected to pollen quality analysis. Except for *J. communis* L. species, represented in the experiment by three natural stands occurring on the localities Priechod, Cervena Skala, and Besnik, the remaining two species originated from one locality each, i.e. *J. sibirica* Lodd. in Burgsd. from Kralova Hola Mtn. and *J. communis* var. *intermedia* (Schur) Sanio from Kralová Studna Mtn. The last mentioned population is regarded as a hybrid form between *J. communis* and *J. sibirica*, amounting to 20–25 shrubs only^3^. Partially, it is also true of the *J. communis* population from Besnik with admixture of some individuals of intermediate habitus. Some characteristics of these localities are given in Table 3. Experimental research and field studies on juniper plants, including collection of plant material, comply with relevant institutional, national, and international guidelines and legislation. Owing to the fact that common juniper (*J. communis*) is not a protected species in Slovakia, and the localities Priechod and Cervena Skala represent public lands, the collection of material for research does not require any specific permission and is done in accordance with the national legislation Act No. 543/2002 Z. z. on conservation of nature and landscape, § 4 and 47 and Act No.220/2004 Z. z. on protection and use of agricultural land. The locality Besnik is an integral part of the National Park Muranska Planina, and the same is true of the localities Kralová Hola and Kralová Studna both of which are involved in the National Park Low Tatras. The pollen collection on the above mentioned localities was made based on permissions issued by the Administration Office in Revuca under No. SNPMP/329-001/2002 on May 5th, 2022 and No. NAPANT/551-001/2022 on May 6th, 2022. The approval of these decisions was released by the District Office in Banska Bystrica, Department of Environmental Care, on May 13th, 2022. In total, 15 male shrubs of each *J. communis* population under study were used in pollen quality estimation. The populations *J. sibirica* and *J. communis* var. *intermedia* were exceptions in this respect, with 14 and 5 shrubs, respectively, involved in pollen collection. Twigs with mature microstrobili were cut at the beginning of pollen shedding, placed in paper bags, and transferred to the laboratory the same day. The next day, the twigs of individual shrubs with opened microsporangia were sieved separately, and the pollen obtained was left for an additional 1–2 h on a sheet to dry more completely. Pollen obtained in this way was subsequently used in the germination test. Pollen samples of individual male shrubs were processed separately. Extracted pollen was stored in glass test tubes that were plugged loosely with cotton-wool caps and placed into a desiccator with silica gel. The desiccator was kept in a refrigerator until the germination test. This portion of samples was attributed to fresh pollen. The period between storing fresh pollen in the refrigerator and its use in the germination test has not exceeded 2 days.

**Table 3:** Altitude and climatic characteristic of the localities under study (source: www.meteoblue.com).

Locality	Altitude (m)	Annual temperature (average—°C)	Annual precipitation (average—mm)
Priechod	530	21.3	50.1
Cervena Skala	785	17.1	86.2
Besnik	994	17.1	86.2
Kralova Hola	1846	3.0	106.2
Kralova Studna	1284	8.7	94.0

### Deep-freezer storage of juniper pollen

This part of study involved the mature pollen of *J. communis* and *J. sibirica* harvested from 6 individuals of each. The same individuals were used in the freshly collected and deep-freezer-stored pollen germination test. Owing to the bad experiences with deep-freezer pollen storage in desiccators made of glass (cracking of glass), the test tubes with juniper pollen were placed in double-walled polyethylene bags with silica gel inside. The sealed bags with pollen were kept at − 81 °C for one year. Two days before the germination test, the deep-freeze stored pollen was shifted to the freezer at − 20 °C and subsequently to the refrigerator at + 4 °C.

### In vitro germination of juniper pollen

Pollen germination was carried out at 25 °C in the dark with a separate evaluation of pollen viability in each shrub under study. The cultivation media consisted of 1.5% agar and 5% sucrose^25^, the latter being reported to be optimal for common juniper pollen germination in culture^7,10^. The pollen was evenly dusted over the gel surface in small Petri dishes (47 mm diameter) by blowing it from a soft-hair brush. Each dish was placed in a larger Petri dish (70 mm diameter) containing 2 ml H_2_O and covered with a lid. Each sample was tested in triplicate. Plates were incubated for 7 days, which was necessary for juniper pollen to fully express the growth potential of pollen tubes^10^. During the second half of the culture period, the cultivation media was contaminated by fungal spores and bacteria on the pollen surface and from air^26^. The number of germinated pollen grains was recorded from a random sample of 100 pollen grains, and the pollen tube length was measured on a sample of 30 pollen grains on each Petri dish (a total of 300 pollen grains and 90 pollen tubes per shrub). Variation in pollen germination rates and pollen tube length was analyzed using analysis of variance (ANOVA) under a partly nested design (factors treatment, population. Individual nested within a population; all factors were considered fixed). Prior to ANOVA, pollen germination rates were arcsine-transformed^27^. Pairwise contrasts were tested using Duncan's tests.

### Pollen dispersion scoring

The dispersion potential of the pollen was followed in *J. communis* only, in its natural locality, using one shrub and pollen trap method. The individual under study is characterized by a relatively solitary occurrence on a clearing with the neighbouring male shrubs growing at least 40 m from the respective individual. The experiment was repeated for two years using the same test shrub but different distances between stakes with fastened microscopic slide traps. In 2021, the stakes were installed at 2, 4 and 6 m distances from the test shrub on the four sides of the shrub. In 2022, the stakes were 10, 17, and 24 m from the test shrub. Each stake possessed a microscopic slide fastened at 90 cm height. The surface of the microscopic slide was oriented towards the test shrub, which was covered with a thin layer of vaseline, serving as a pollen trap.

## Data Availability

Original data obtained by microscopic measuring of pollen characteristics are available in K.A. coauthor on nrgrkorm@savba.sk and M.G. corresponding author on galgoci7@gmail.com.
